# Mannoprotein Cig1 contributes to the immunogenicity of a heat-killed F-box protein Fbp1 *Cryptococcus neoformans* vaccine model

**DOI:** 10.1128/iai.00355-25

**Published:** 2025-11-28

**Authors:** Samantha L. Avina, Siddhi Pawar, Roshni N. Kadam, Amariliz Rivera, Chaoyang Xue

**Affiliations:** 1 School of Graduate Studies at the Health Science Campus, New Jersey Medical School, Rutgers University Newark 67206 , Newark, New Jersey, USA; 2 Public Health Research Institute, and Department of Microbiology, Biochemistry, and Molecular Genetics, New Jersey Medical School, Rutgers University 67206 , Newark, New Jersey, USA; 3 Center for Immunity and Inflammation, and Department of Pediatrics New Jersey Medical School, Rutgers University Newark 67206 , Newark, New Jersey, USA; University of California Davis, Davis, California, USA

**Keywords:** HK-*fbp1*, fungal vaccine, mannoprotein, Cig1, murine model, immunogenicity

## Abstract

Currently, no fungal vaccine exists for clinical use, while fungal infections are responsible for over 1.5 million deaths every year. Our previous studies identified a *Cryptococcus neoformans* mutant strain *fbp1*Δ as a potential vaccine candidate. This strain contains deletion of the F-box protein Fbp1, a key subunit of the SCF E3 ligase complex necessary for ubiquitin-mediated proteolysis. Vaccination with heat-killed *fbp1*Δ (HK-*fbp1*) can elicit an interferon gamma (IFN-γ)-dependent Type 1 immune response and provide protection against *C. neoformans* and its sibling species *C. gattii*. However, we have yet to decipher the immunogenic factor(s) expressed by the *fbp1*∆ mutant that are responsible for the induction of the protective immune response. In this study, we have identified that the capsule plays an important role in HK-*fbp1* vaccine-mediated protection as acapsular HK-*fbp1* cells showed diminished protection against wild-type challenge. Additionally, our studies have shown that Cytokine Inducing Glycoprotein 1 (Cig1), a GPI-anchored mannoprotein, is regulated by Fbp1 and contributes to the immunogenicity of HK-*fbp1*. Deletion of Cig1 in the *fbp1*Δ background resulted in decreased recruitment of antifungal effector T cells and diminished production of protective inflammatory cytokines by the host. Furthermore, loss of Cig1 in the *fbp1*Δ mutant resulted in reduced protection in vaccination survival studies at lower vaccine inoculum doses compared to HK-*fbp1*. In aggregate, these findings demonstrate Cig1 is an antigen contributing to the immunogenicity of HK-*fbp1* that may be utilized to further optimize the HK-*fbp1* fungal vaccine as a tool in the arsenal against invasive fungal infections.

## INTRODUCTION

*Cryptococcus neoformans* is an encapsulated yeast that is the primary causative agent of cryptococcosis, which predominantly affects immunocompromised populations. *C. neoformans* has been identified as the leading fungal pathogen responsible for fungal encephalitis and is responsible for ~20% of AIDS-related deaths worldwide (1). In 2022, the inaugural World Health Organization fungal priority pathogens list ranked *C. neoformans* in the top research critical priority group of fungal pathogens as cases of invasive fungal disease continue to rise (2). External factors that contribute to increased susceptibility of *C. neoformans* include increased use of immunosuppressants (i.e., cancer chemotherapies and organ transplant recipients) and potentially climate change as global temperatures rise (3, 4). Invasive fungal pathogens like *C. neoformans* are eukaryotes that share large similarities of cellular machinery with human hosts, making treatment options limited, prolonged, and often unsuccessful. As *C. neoformans* continues to be a rising global health concern, the greatest limit to the antifungal arsenal is lack of antifungal vaccination strategies. Currently, no fungal vaccines are available for clinical use.

Protection against *C. neoformans* is primarily driven in a CD4^+^ T cell-dependent manner. HIV/AIDS immunocompromised populations deficient in CD4^+^ T cells are highly susceptible to cryptococcosis disease and thus emphasize the importance of T cell-mediated immunity in the context of the host response against cryptococcal infection (5). Humoral immunity is also understood to contribute to host immunity as previous studies have shown antibodies generated against capsule components GXM and Gal-XM ameliorate fungal burden when administered to murine models but not sufficient in conferring protection against *C. neoformans* challenge in murine models (6–8). More importantly, a balance of inducing a Th1 protective inflammatory response while not generating a detrimental over-inflammatory response is key in the protection of different vaccine strain candidates. In multiple *C. neoformans*-based vaccine candidates, the ability to elicit production of IFN-γ by the host CD4^+^ T or CD8^+^ T cells in the absence of CD4^+^ T cells upon vaccination and subsequent challenge has been critical (9–16).

In our previous studies, we have identified a *C. neoformans* mutant strain lacking F box protein 1 (*fbp1*Δ) as a potential vaccine candidate. The F-box protein is a part of the E3 ubiquitin ligase complex responsible for ubiquitin-mediated proteolysis of target proteins by the 26S proteasome in eukaryotic systems that are required for proper cellular functions. It has been established that while Fbp1 is not required for development of classical virulence factors (melanin, chitin/chitosan, capsule size, etc.) (17), it does regulate cell size, sexual reproduction, and is essential for fungal virulence (18–20). Interestingly, the *fbp1∆* mutant infection triggered strong protective Th1 immunity, and this protective immunity remains even in animals immunized with the heat-killed fbp1 cells (HK-*fbp1*). Therefore, HK-*fbp1* has been developed as an excellent vaccine candidate. However, the immunogens in the HK-*fbp1* vaccine remain uncharacterized.

Cryptococcal mannoproteins are heavily glycosylated and reside in the capsule closer to the cell wall (21, 22) that are highly immunogenic antigens and can stimulate Th1 responses of CD4^+^ T cells to produce cytokines including IL-6, IL-10, IFN-γ, and TNF-α (23–25). Mannosylation of cryptococcal mannoproteins also enhances their immunogenicity for mannose receptors for uptake and presentation to T cells (26–28). Mannoproteins MP-98 and MP-88 have been described to reside close to the cell wall rather than as integral parts of the capsule and largely contribute as immunogens that can be secreted from the cell to trigger immunogenic responses (21, 24, 29, 30). Mannoprotein-deficient strains, including the chitin deacetylase triple mutant (*cda1*Δ *cda2*Δ *cda3*Δ), have been shown to be critical to enhancing its immunogenicity and utilized as a promising *C. neoformans* vaccine candidate (14, 31–34). Yet, studies have also found that cryptococcal mannoproteins, like Krp1, contribute to capsule structuring mainly by subtle altering the distribution of glycans in the cell wall (35). Whether mannoproteins play a role in the immunogenicity of all current cryptococcal-based fungal vaccine candidates has yet to be thoroughly explored.

In this study, we set out to investigate the role of mannoproteins and capsule in mediating the immunogenicity of the heat-killed *fbp1*Δ (HK-*fbp1*) vaccine protection. Here, we report that the capsule plays an important role in HK-*fbp1* vaccine-mediated protection as acapsular HK-*fbp1* cells showed diminished protection against wild-type challenge. Additionally, serum from HK-*fbp1-*vaccinated mice was shown to bind to distinct regions of the capsule in *fbp1∆* strains versus parental H99 strain, indicating the importance of immunogenic antigens in the capsule specific to *fbp1*Δ. We found that Cytokine Inducing Glycoprotein 1 (Cig1, CNAG_01653), a GPI-anchored mannoprotein with a PEST domain sequence, is upregulated in *fbp1*Δ and elicits antibody production in HK-*fbp1* vaccination sera. Furthermore, we found that loss of Cig1 in the *fbp1*Δ background diminishes the protective host response upon vaccination and induction of Th1 response in live strain infection. While differences in protection were only observed at lower vaccine inoculum doses, these studies suggest Cig1 is an immunogenic antigen upregulated in *fbp1*Δ, which contributes to the enhanced immunogenicity of the HK-*fbp1* vaccine and may be a substrate regulated by *fbp1*∆.

## RESULTS

### Capsule production contributes to HK-*fbp1* vaccine protection against H99 *Cryptococcus* challenge

**Fig 1:**
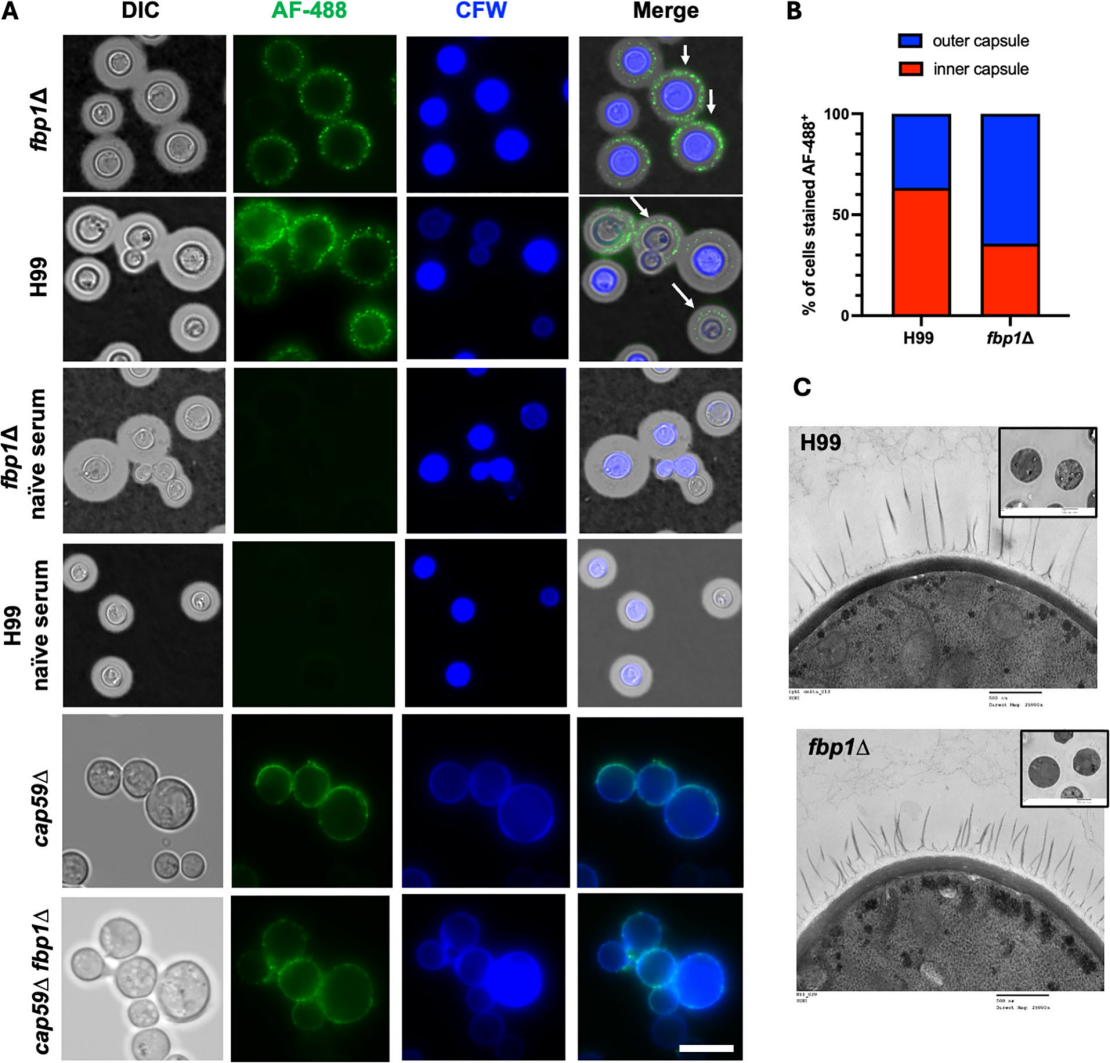
HK**-***fbp1-*vaccinated mouse antibodies bind to different regions of the *fbp1***∆** *Cn* capsule. (**A**) Wild-type H99, *fbp1∆*, *cap59∆, and cap59∆ fbp1*∆ grown in the capsule inducing minimal media for 3 days at 30°C and incubated with HK-*fbp1-*vaccinated mouse serum conjugated to AF-488. Cells were imaged in India ink to show the capsule and stained with Calcofluor White to contrast the fungal cell wall at 100x magnification. Cells were also incubated with serum from naïve mice as control. Images taken in DIC GFP and DAPI channels were overlayed and merged to show distinction of binding by antibodies in vaccinated serum. Bar = 10 μm. (**B**) Quantitative ratio of binding on the inner capsule versus the outer capsule in percentage of total cells counted as AF-488-positive. (**C**) TEM imaging of H99 and *fbp1*∆ comparing the cell wall and capsule structures. Bar = 500 nm.

To determine what antigens contribute to the immunogenicity of *fbp1*Δ*,* we first examined the role of the capsule as it is the first cellular surface that encounters the host and masks cell wall antigens. In previous studies, we broadly screened for differences in classical virulence factors of *fbp1*Δ and saw no differences in capsule size or secreted GXM (17). In TEM imaging comparing H99 and *fbp1*Δ*,* we also saw no major architectural changes between the strains (Fig. 1C). To ascertain whether there are specific regions of the capsule that are immunogenic, we utilized HK-*fbp1-*vaccinated mouse serum in an immunofluorescence assay to visualize and compare the localization of HK-*fbp1* vaccine antibody binding. Interestingly, there was a difference in HK-*fbp1* serum binding to the capsule of *fbp1*Δ compared to H99 (Fig. 1A). Specifically, serum from HK-*fbp1-*vaccinated mice bound to the outer rim of the capsule in *fbp1*Δ compared to H99, and a higher percentage of *fbp1*Δ cells showing serum binding localized to the outer region of the capsule (Fig. 1B). Due to previous exposure to HK-*fbp1* Cryptococcal antigens, we anticipated serum from HK-*fbp1-*vaccinated mice would produce an array of antibodies to many regions of the Cryptococcal cell. We, therefore, also tested the antibody binding in an acapsular strain *cap59*∆ and the *cap59*∆ *fbp1*∆ double mutant. While there was an observed difference in localization, antibodies still bound robustly to the acapsular *cap59*∆ strains (Fig. 1A). As antibodies from HK-*fbp1* serum bound distinctively to acapsular strains, we desired to confirm whether the capsule is required for HK-*fbp1* protection.

**Fig 2:**
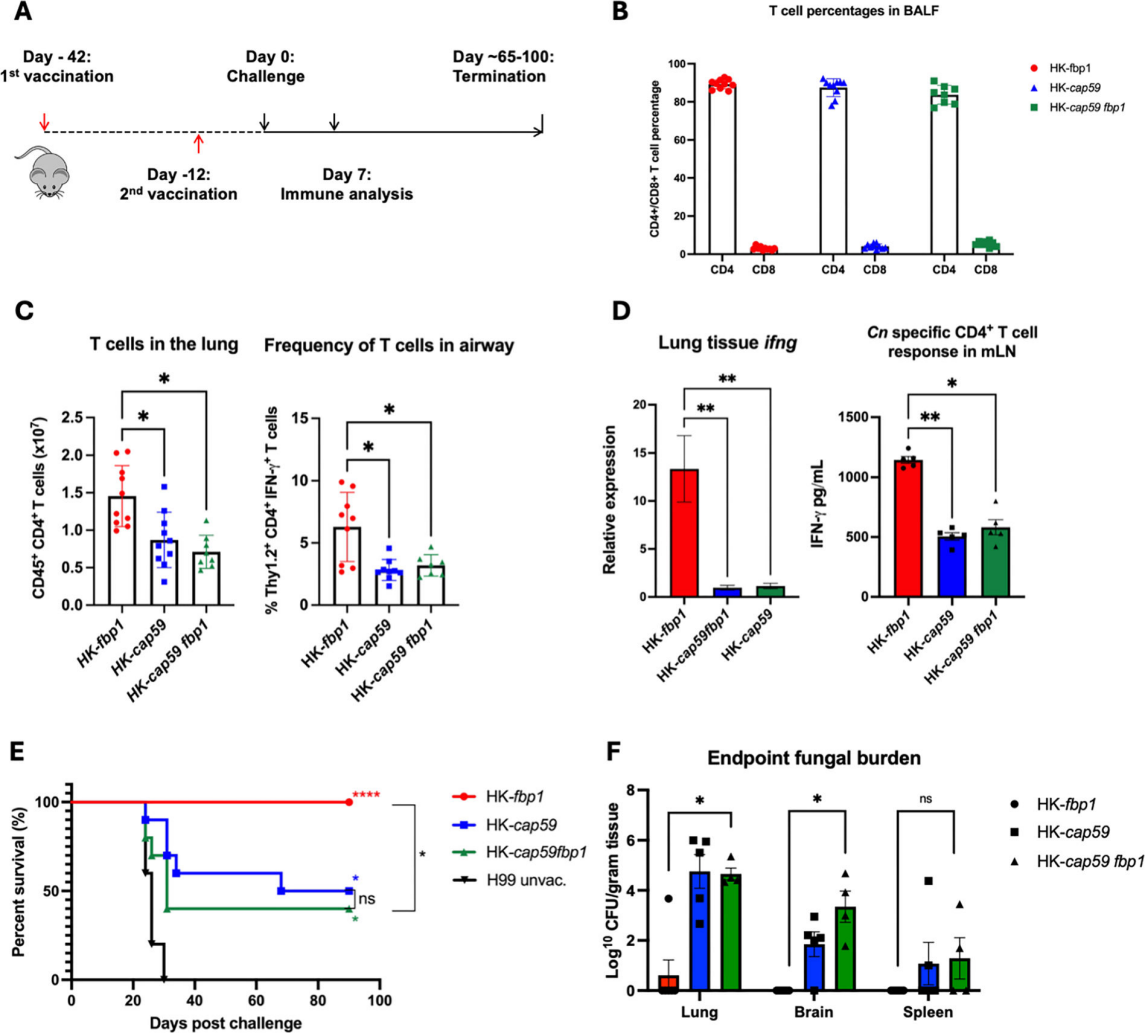
Capsule is required for full HK**-***fbp1* vaccine-mediated protection. (**A**) Schematic of the vaccination strategy. Mice are vaccinated with HK-*fbp1* or acapsular strains (HK-*cap59* or HK-*cap59 fbp1*) 42 days and 12 days before challenge with 1 × 10^4^ H99 wild-type strain. (**B–D**) Day 7 post-H99 challenge immune response. IFN-γ producing CD4^+^ T cells in BALF (**B**), IFN-γ cytokine readout from isolated T-cells of vaccinated mice presented H99 antigen (**C**), qPCR from total lung tissue and ratio of CD4^+^ to CD8^+^ T cells in the vaccinated cohorts (**D**). *P < 0.05, **P < 0.001, and ****P < 0.0001, Kruskal-Wallis test with Dunn’s multiple comparison test. (**E–F**) Survival curve of mice vaccinated with HK-*fbp1* or acapsular strains (**E**) and fungal burden (**F**) of remaining survivors. *P < 0.05; ****P < 0.0001 (log rank [Mantel-Cox] test). Data are representative of two independent experiments.

We next used the acapsular *fbp1*Δ strain (*cap59*Δ *fbp1*Δ) to investigate the adaptive immune response in the context of vaccination compared to *fbp1*D. In this vaccination experiment utilizing the previously established intranasal vaccination method (Fig. 2A) to compare heat-killed acapsular strains HK-*cap59 fbp1* and HK*-cap59*, we observed that mice vaccinated with either heat-killed acapsular strain showed similar CD4^+^ and CD8^+^ T cell population percentages in the bronchiolar lavage fluid (BALF) (Fig. 2B). However, heat-killed acapsular strain-vaccinated cohorts showed diminished recruitment of CD4^+^ T cells in the lung compared to capsular HK-*fbp1* (Fig. 2C). Furthermore, the functionality of CD4^+^ cells from the BALF to produce key protective cytokine IFN-γ was also diminished, as observed via flow cytometry (Fig. 2D and Fig. S1). Survival studies showed that heat-killed acapsular strain-vaccinated cohorts showed a significant decrease in survival compared to the capsular HK-*fbp1* and succumbed to lethal cryptococcal meningitis (Fig. 2E and F). These findings supported that while the capsule is required for full HK-*fbp1-*mediated protection, it is not sufficient as 40% of the acapsular HK-*fbp1* strain-vaccinated cohorts still survived until the termination of the survival (endpoint) at day 70 post-challenge. In the remaining survivors, fungal dissemination in the lung and brain was present, indicating these cohorts may have eventually succumbed to cryptococcal infection. These results suggest that the capsule is required but not sufficient in inducing HK-*fbp1-*mediated immune protection. Thus, we proceeded to assess the role of non-GXM- or GalXM-based antigens.

### Cytokine inducing glycoprotein 1 (CIG1) is overexpressed in *fbp1*D and a substrate of *Fbp1* localized to the cell wall.

**Fig 3:**
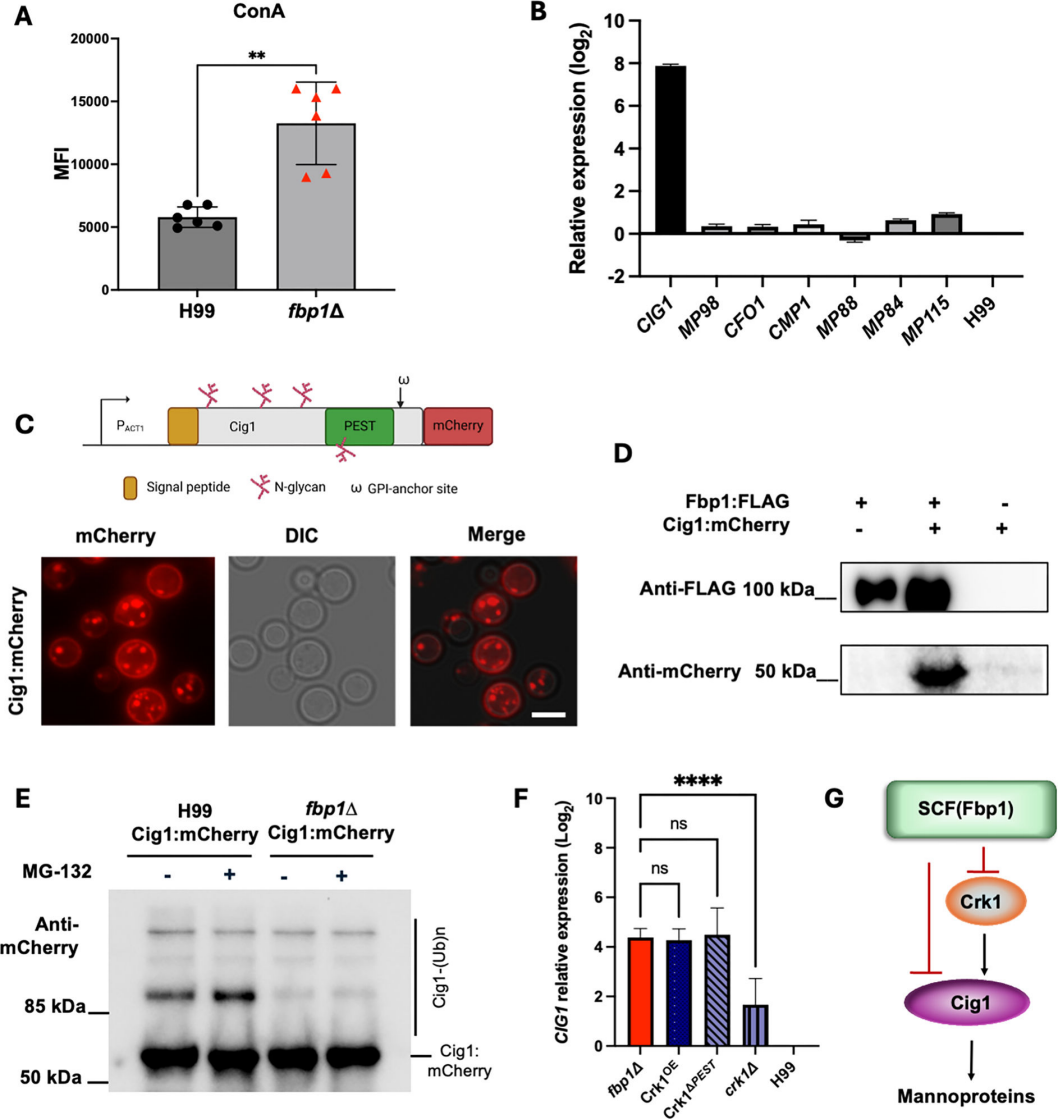
Cig1 interacts with Fbp1 and is overexpressed at the transcriptional level in *fbp1***∆**. (**A**) Comparison of MFI ConA-FITC-positive stained cells amongst mutant strains. **P < 0.0005, nonparametric Mann Whitney test. Data are cumulative of two independent experiments. (**B**) Expressions of *CIG1, CMP1, CFO1, MP88, MP84, MP98, MP115,* and *GAPDH* analyzed by qRT-PCR assay. Gene expression levels were normalized using the endogenous control gene *GAPDH* and analyzed using ∆∆CT methods. (**C**) Diagram of Cig1:mCherry fusion construct generation and Cig1 protein putative N-glycosylation sites (red chains), PEST domain predicted region (green box), and a transmembrane domain region (W). Cig1 is fluorescently tagged with an mCherry protein and localized to the cell membrane. (**D**) Co-immunoprecipitation of Fbp1:FLAG with Cig1:mCherry from strain lysates expressing only Fbp1:FLAG, Cig1:mCherry, or both Fbp1:FLAG and Cig1:mCherry. Immunoprecipitated samples were immunoblotted with either anti-FLAG (top) or anti-mCherry (bottom). (**E**) *In vivo* ubiquitination assay to confirm Cig1 is a substrate of Fbp1 E3 ligase. Total proteins from H99 and *fbp1*∆ strains expressing Cig1:mCherry with or without proteasome inhibitor MG-132 treatment were purified using anti-mCherry antibody pull-down and detected in a Western blot. The high-molecular weight bands indicate poly-ubiquitinated Cig1 proteins (Cig1-(Ub)n). (**F**) Expression of *CIG1* in multiple listed strain backgrounds analyzed by qRT-PCR assay. Gene expression levels were normalized using the endogenous control gene *GAPDH* and analyzed using the ∆∆CT method. **** *P* <0.0001. One-way ANOVA with multiple comparisons. Ons, no significance. (**G**) A proposed model of Cig1 regulation by the SCF(Fbp1) E3 ligase.

Because mannoproteins are known cell surface immunogens important for host immune recognition (21, 23, 36), and we did not detect any difference in chitin and chitosan levels in the *fbp1*∆ cells, we decided to investigate the production of mannoproteins in H99 and its *fbp1*∆ cells by concanavalin A (ConA) staining. Although ConA can also bind to D-glycosyl groups on glycoproteins, its staining has been commonly used to measure the surface mannose level in fungal cells due to its higher binding affinity for mannose than glucose (37, 38). We found that *fbp1*∆ cells showed higher FITC-ConA signal intensity in comparison to H99 (Fig. 3A)**,** suggesting an overproduction of mannoproteins in the mutant. To identify the genes responsible for the difference in mannoprotein, we screened eight reported mannoprotein encoding genes using qRT-PCR under YPD growth conditions. Our data demonstrated that one of them, Cytokine Inducing Glycoprotein 1 (*CIG1*, CNAG_01653), was highly upregulated in the *fbp1*∆ background (Fig. 3B). Interestingly, in our previous Fbp1 pull-down studies using a ligase-trapping system, we also identified Cig1 as one of Fbp1-interacting proteins as potential SCF(Fbp1) substrates (unpublished). We posit that Cig1 may be a substrate of Fbp1 that can undergo post-translational modifications; at the same time, it is also transcriptionally regulated by Fbp1, either directly or by modulation of other downstream substrates. These studies suggest that Cig1 may be an immunogen overproduced in *fbp1*∆ cells. Therefore, we decided to further analyze the potential role of Cig1 in *fbp1*∆-mediated immune activation. Cig1 is a heavily glycosylated GPI-anchored mannoprotein (Fig. 3C). It has been identified as a hemophore required for iron nutrient sensing and uptake for *C. neoformans* in host conditions (39). Excitingly, while we were analyzing the Cig1 regulation by Fbp1 E3 ligase, it was recently independently reported that Cig1 is an antigen in *C. neoformans* Znf2^OE^ vaccine that can be utilized for mRNA-based vaccine development (40). This independent study further supported our hypothesis that Cig1 may be an important immunogen in the HK-fbp1 vaccine model. To confirm that Cig1 interacts with Fbp1, we generated a C-terminus mCherry-tagged *CIG1* construct under the control of the *C. neoformans* actin promoter (*P_ACT1_-CIG1:mCherry*) and transformed it into a *C. neoformans* strain expressing Fbp1:FLAG (Fig. 3C). The expression of Cig1 was confirmed by mCherry expression. We found that the mCherry signal was primarily localized to the plasma membrane, consistent with its function as a GPI-anchored mannoprotein (Fig. 3C). We conducted a co-immunoprecipitation (Co-IP) experiment using this *C. neoformans* strain expressing both Fbp1:FLAG and Cig1:mCherry proteins and confirmed the interaction between Fbp1 and Cig1 (Fig. 3D). Cig1 has a PEST domain, a putative signature of short half-life proteins often subjected to E3 ligase-mediated ubiquitination (41, 42), further indicating that Cig1 is likely a substrate of the Fbp1 and subjected to Fbp1-mediated ubiquitination and degradation. To confirm the Cig1 ubiquitination, we expressed Cig1:mCherry fusion protein in both wild-type and *fbp1*∆ strain background and conducted an *in vivo* ubiquitination assay following a previously developed protocol (18). The Cig1:mCherry protein was purified by immunoprecipitation using anti-mCherry antibody, and Cig1 ubiquitination was observed as high-molecular weight Cig1 protein bands in a Western blot. Our assay revealed that Cig1 is ubiquitinated, and its ubiquitinated protein signal decreased in the *fbp1*∆ background (Fig. 3E). These data confirmed that Cig1 can be ubiquitinated by Fbp1 E3 ligase.

These studies confirmed that Cig1 is a substrate of Fbp1 that can undergo post-translational modifications. However, it did not address how Fbp1 also transcriptionally regulates *CIG1* expression. In our previous studies, we identified that Crk1, a CDK-related kinase 1, is a direct downstream substrate of Fbp1 (18). To test the hypothesis that Fbp1 may regulate *CIG1* expression through its regulation of the Crk1 protein, we measured the *CIG1* expression in wild-type, *crk1*∆, and CRK1 overexpression (*CRK1^OE^*) allele expressing strains. Our qRT-PCR assay showed that *CIG1* expression is significantly induced in *CRK1* overexpression strains, including *CRK1*^ΔPEST^ and *CRK1^OE^* backgrounds, at a level similar to that of *fbp1*, and reduced in the *crk1*∆ background, indicating that *CIG1* expression is positively regulated by Crk1 (Fig. 3F). Hence, our data support the model that Fbp1 regulates Cig1 at two different levels. Namely, Fbp1 suppresses *CIG1* gene expression by degrading its substrate Crk1, and then Fbp1-mediated Cig1 ubiquitination also promotes its degradation (Fig. 3G). Such dual-level regulation also suggests a tight functional association between Fbp1 and Cig1 in modulating the cellular function, leading to significant overproduction of Cig1 in the *fbp1*∆ background.

### Cig1 is required for fungal cell wall integrity and macrophage interaction.

**Fig 4:**
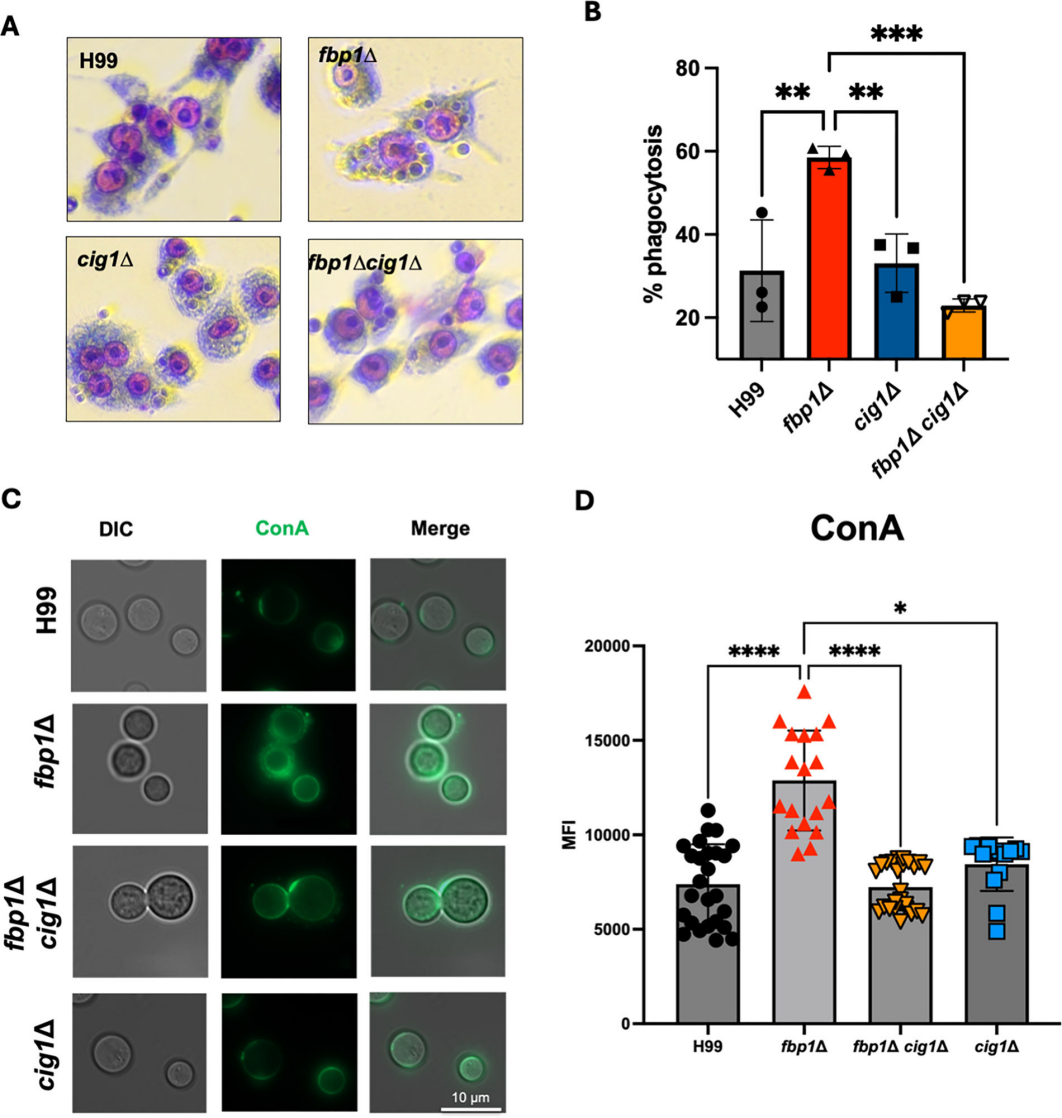
Cig1 mannoprotein alters mannose presence in the cell wall and phagocytosis in *fbp1*Δ background. (**A**) 5 × 10^4^ activated RAW26 macrophage cells incubated with 2 × 10^5^ opsonized *C.n* for 2 h. Percentage of phagocytosis of *C.n* with representative images shown at 20x magnification. (**B**) Qualification data of the percentage of yeast cell engulfed by macrophages to determine the phagocytosis efficiency *P < 0.05, **P < 0.005, and ***P < 0.0005, One-way ANOVA with Dunnett’s multiple comparisons test. Data are representative of two independent experiments. (**C**) Comparison of MFI ConA-FITC-positive stained cells among mutant strains. (**D**) Quantification of the ConA staining signal for each listed strain. *P < 0.05; ****P < 0.0001, Kruskal-Wallis test with Dunn’s multiple comparison test. Data are cumulative of six independent experiments.

Consistent with previous findings, the *cig1*∆ single mutant did not show any clear phenotypic defect compared to the wild-type. We then constructed an *fbp1*Δ *cig1*Δ double mutant to characterize the phenotype of *fbp1*Δ in the context of Cig1 deletion. Classical phenotypic characterization of the *fbp1*∆ *cig1*∆ mutant strains revealed only modest phenotypic differences compared to the parental *fbp1*Δ on the cell wall and membrane integrity (Fig. S2). The *fbp1*Δ *cig1*Δ strain showed growth inhibition on Congo Red and L-DOPA agar media when incubated at 37°C. No inhibition was observed when the same plates were incubated at 30°C. In an *in vitro* phagocytosis assay using macrophage-like cell line J774, the *fbp1*Δ *cig1*Δ cells showed a decrease in phagocytosis when compared to the *fbp1*Δ single mutant, to a level similar to that of H99 (Fig. 4A and B). Furthermore, the *cig1*Δ mutant also showed low percentages of phagocytosis resembling wild-type H99 compared to *fbp1*Δ (Fig. 4B). These *in vitro* assays and phenotypic characterizations show that Cig1 moderately alters *fbp1*Δ growth in different stress conditions (i.e., cell wall integrity and melanin production) and initial interactions with host immune cells.

To confirm that loss of Cig1 indeed alters *fbp1*Δ mannose levels, we stained all mutants with ConA and compared mutants via flow cytometry analysis of ConA-positive stained cells. This experiment showed that *fbp1*Δ did have higher mannose levels compared to H99 and the *cig1*Δ mutant (Fig. 4C and D). Furthermore, the loss of a high ConA positive signal in the *fbp1*Δ *cig1*Δ double mutant revealed Cig1 is a contributor to high mannose presence in the *fbp1*Δ mutant.

### Loss of Cig1 in *fbp1*Δ diminishes induction of protective Th1/Th17 immune response.

**Fig 5:**
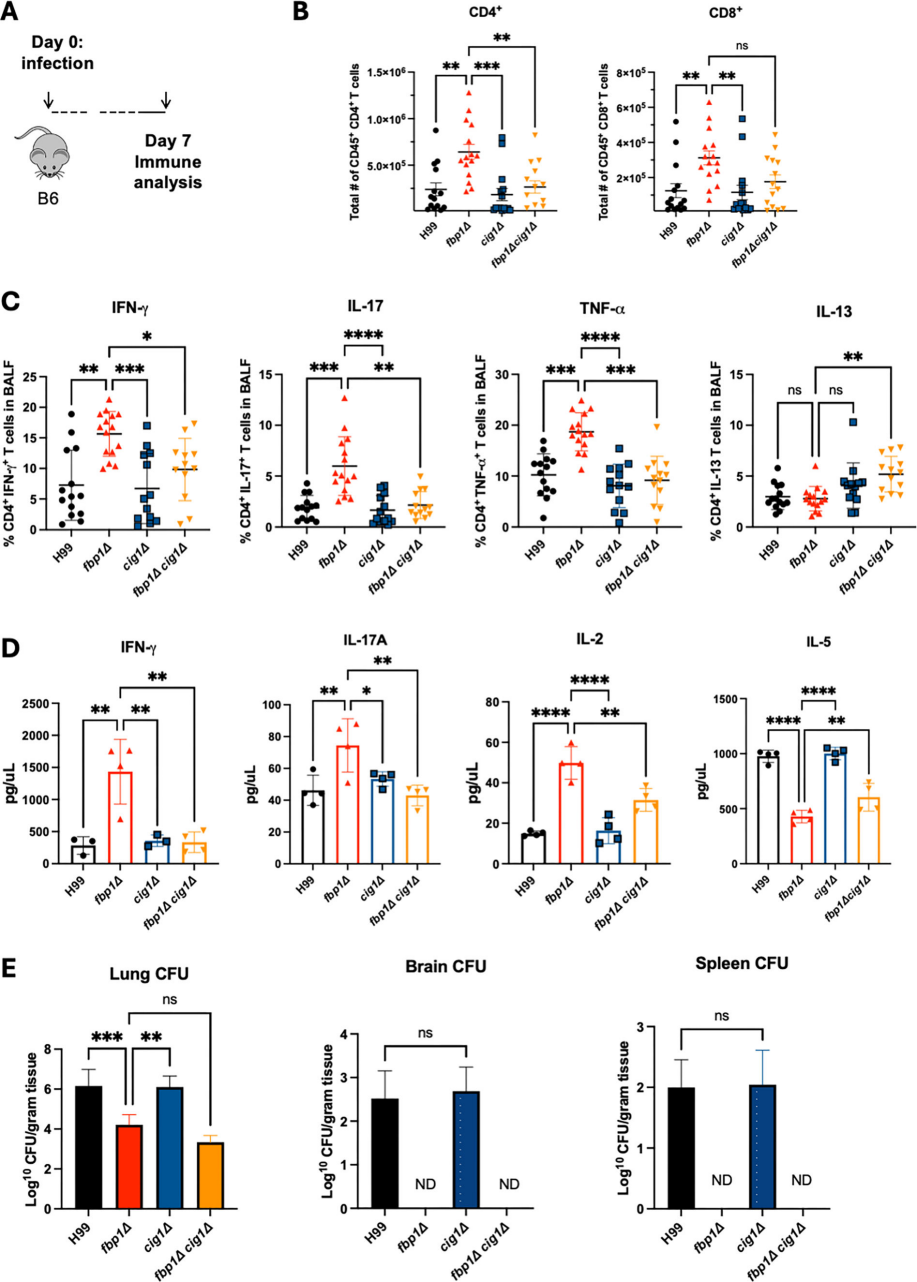
Loss of Cig1 in *fbp1∆* background diminishes Th1 and Th17 responses at day 7 post infection compared to parental *fbp1∆* strain. (**A**) Schematic of infection with live strains at 10^6^ (*fbp1*∆, *fbp1*∆ *cig1*∆) and 10^5^ (H99, *cig1∆*) in B6 mice (*n* = 4 or 5 per group). (**B**) CD4^+^ and CD8^+^ T cell populations in lung at day 7 post-infection. (**C**) Cytokine readout from CD4^+^ T cells in BALF 7 days post-infection. Kruskal-Wallis test with Dunn’s multiple comparison test was used for both B and C data sets. *P < 0.05, **P < 0.001, ***P < 0.0005, and ****P < 0.0001. (**D**) Cytokine readout from mLN purified CD4^+^ T cells stimulated with H99 antigen for 48–72 hr. (**E**) Fungal CFUs for lung, brain, and spleen of mice day 7 post-infection. One-way ANOVA with Dunnett’s multiple comparisons test was used for data analysis in **D** and **E**. *P < 0.05, **P < 0.005, and ***P < 0.0005. ns, no significance; ND, not detectable. Data are representative of three independent experiments.

To determine the role of Cig1 in *fbp1*Δ-mediated host immune response during live infection, we infected murine cohorts comparing *fbp1*Δ and *fbp1*Δ *cig1*Δ to analyze T cell populations and function (Fig. 5A). Both mutant strain-infected cohorts were administered higher inoculums (10^6^ CFU/mouse) than H99 and *cig1*Δ (10^5^ CFU/mouse) controls to reach a comparable fungal burden in the infected lungs for immunological comparison as the *fbp1*Δ parental strain has significantly reduced lung CFU after 3 day post-inoculation, as described in our previous publications (17). Compared to *fbp1*Δ, the mice infected with *fbp1*Δ *cig1*Δ cells had diminished CD4^+^ T cell recruitment in the lungs and significantly reduced production of key protective cytokines (i.e., IFN-γ, IL-17A, and TNF-α) in the airways (Fig. 5B and C). To further confirm these functional differences, we performed complementary CD4^+^ T cell recall experiments utilizing primed T cells from the mediastinal lymph nodes (mLN) presented with H99 antigen and measured cytokine production differences. Similar to our flow cytometric data, *fbp1*Δ *cig1*Δ-infected animals produced lower key protective cytokine levels compared to *fbp1*Δ (Fig. 5D). However, when assessing fungal burden differences 7 days post-infection, both *fbp1*Δ and *fbp1*∆ *cig1*∆ mutant strains had low fungal burden compared to H99 and *cig1*Δ (Fig. 5E). These findings suggest loss of Cig1 altered the host immunological response to *fbp1*Δ infection but did not change the avirulence of *fbp1*Δ that inhibits fungal dissemination.

### HK-*fbp1* primed CD4^+^ T cells elicit protective cytokine responses to Cig1 isolated antigen and contribute to HK-*fbp1* vaccination-mediated host protection

**Fig 6:**
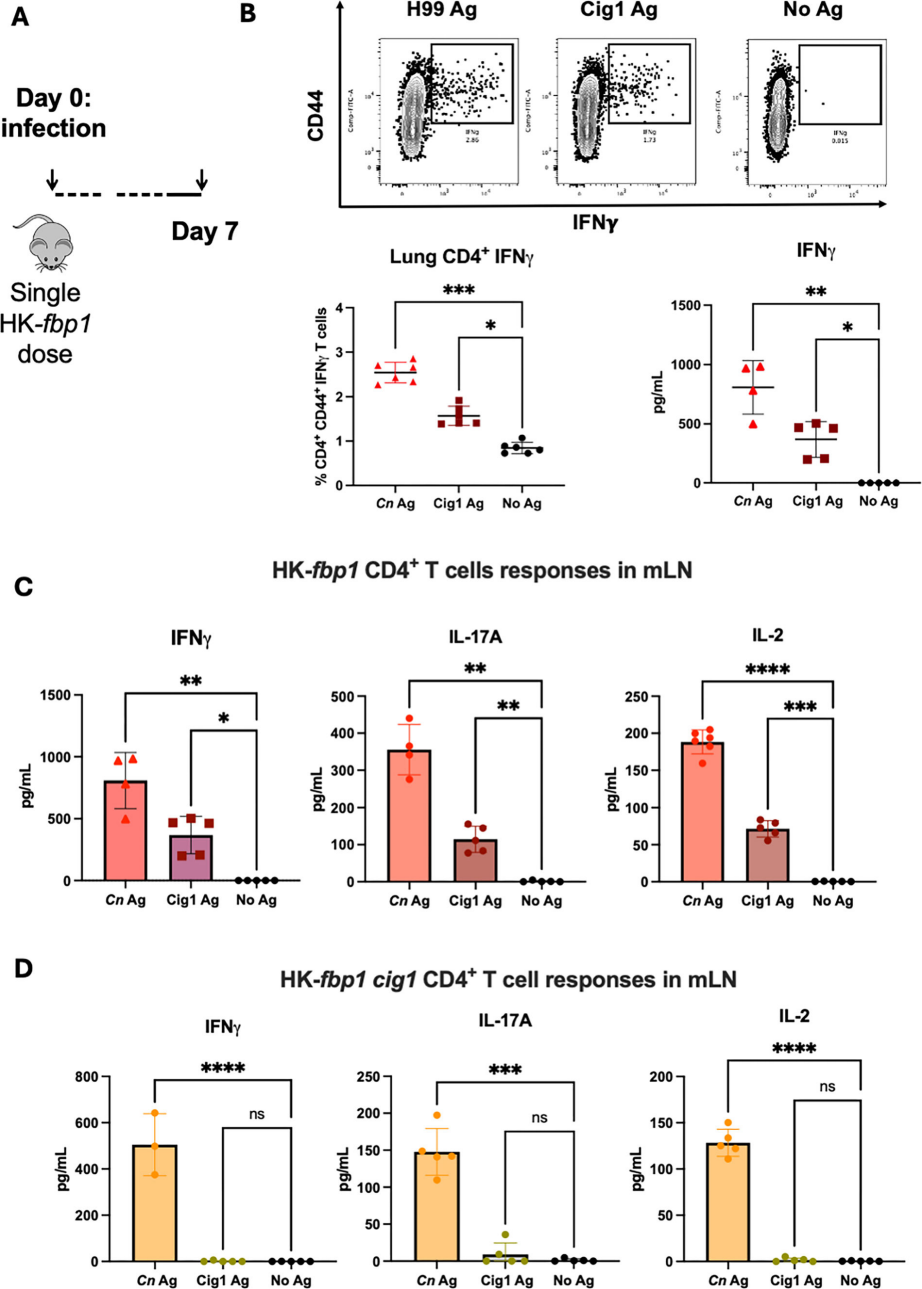
HK**-***fbp1-*primed lymphocytes respond to Cig1 as a stimulating immunogen. (**A**) Schematic of the short-term vaccination model. Balb/C mice (*n* = 7–10) are intranasally administered one dose of 5 × 10^7^ HK-*fbp1* cells and sacrificed 7 days post vaccination. (**B**) Top panels flow plots are representative of IFN-γ producing HK-*fbp1* primed CD4^+^ T cells from whole lung suspension after *ex vivo* restimulation with either whole H99 sonicated antigen, purified Cig1 antigen, or no antigen. Bottom panels show quantification results of the percentage of IFN-γ producing CD4^+^ T cells and concentration of IFN-γ produced by HK-*fbp1* T cells from mLN primed with *Cn* antigen or purified Cig1 antigen. **C–D**. Comparison of IFN-γ, IL-17A, and IL-2 production by HK-*fbp1* (**C**) or HK-*fbp1 cig1* (**D**) primed mLN CD4^+^ T cells after *ex vivo* restimulation with either whole H99 sonicated antigen, purified Cig1 antigen, or no antigen. *P < 0.05, **P < 0.005, ***P < 0.0005, and ****P < 0.0001, One-way ANOVA with Dunnett’s multiple comparisons test. Data are representative of two independent experiments.

To further dissect the importance of Cig1 in *fbp1*Δ immunogenicity, we vaccinated murine cohorts with HK-*fbp1* and assessed whether HK-*fbp1* primed CD4^+^ T cells responded to purified Cig1 antigen (Fig. 6A and Fig. S3). HK-*fbp1-*primed single-cell suspension lung tissue samples were restimulated with either whole-cell H99 sonicated antigen, purified Cig1 antigen, or no antigen *ex vivo* and assessed for IFN-γ production by activated CD4^+^ T cells via flow cytometry (Fig. S4). Results showed that HK-*fbp1-*vaccinated CD4^+^ T cells in the lung environment did produce a protective IFN-γ cytokine response to Cig1 antigen compared to the no antigen controls (Fig. 6B). Furthermore, sonicated H99 antigen stimulated HK*-fbp1 cig1* cohort showed lower levels of CD4^+^ IFN-γ producing cells compared to HK*-fbp1*. To further confirm HK-*fbp1* primed CD4^+^ T cells response to Cig1 antigen, we conducted T cell recall experiments and confirmed Cig1 antigen can elicit a protective cytokine response (IFN-γ, IL-17A, and IL-2) in HK-*fbp1-*primed T cells from the mLN, whereas HK-*fbp1 cig1* primed CD4^+^ T cells did not (Fig. 6C and D).

**Fig 7:**
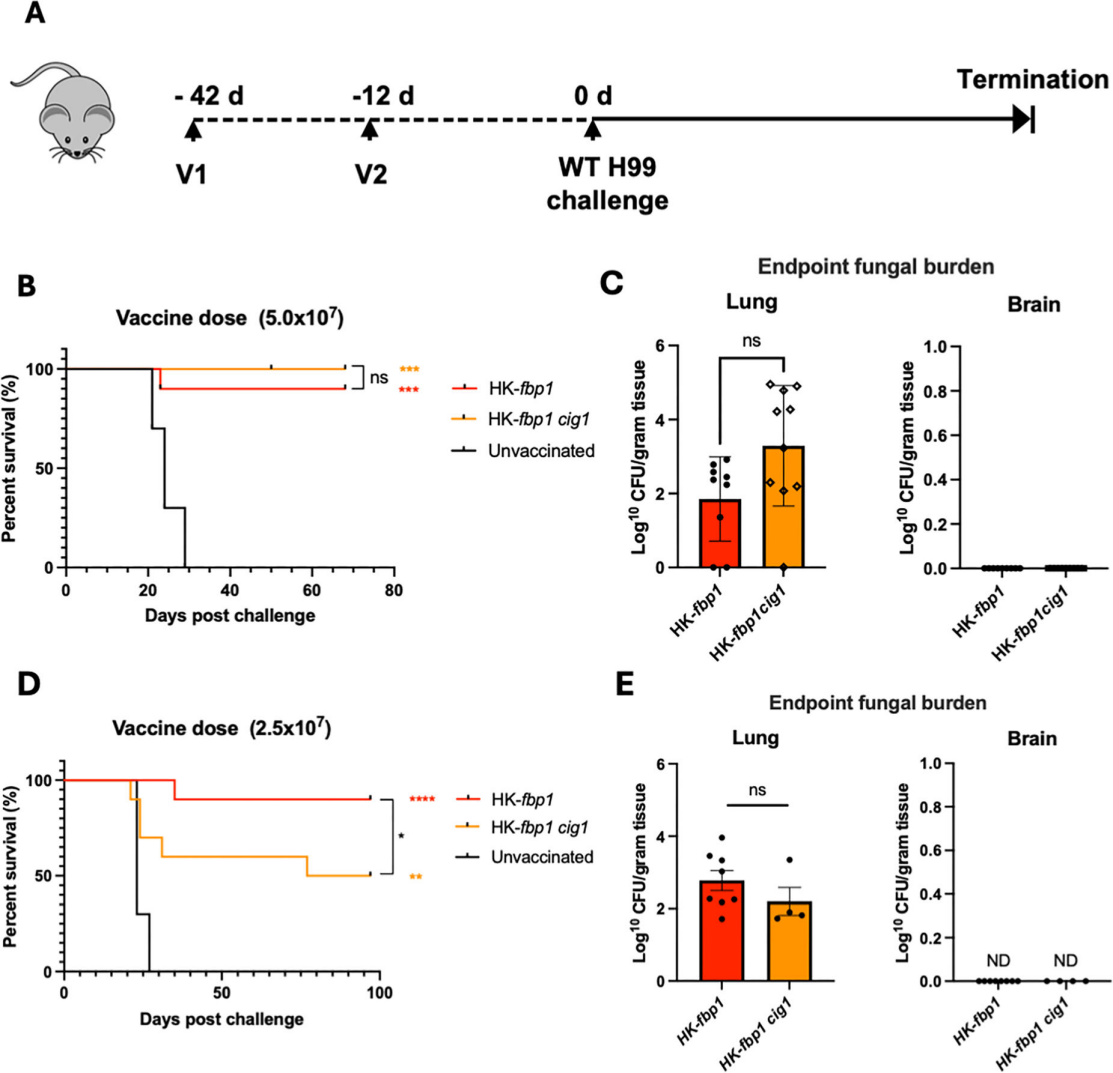
Cig1 plays a role in HK**-***fbp1* protection observed only at half-dose vaccination. (**A**) Schematic of the vaccination strategy. Mice are vaccinated with HK-*fbp1* or HK-*fbp1 cig1* strains twice before challenged with 1 × 10^4^ H99 wild-type strain. (**B and C**) Survival rate of mice vaccinated with HK-*fbp1* or HK-*fbp1cig1* strains at full dose (5 × 10^7^ cells/mouse) and challenged with H99 (**B**) and the fungal burden of remaining survival animals at the endpoint of the experiment (**C**). (**D and E**) Survival rate of mice vaccinated with HK-*fbp1* or HK-*fbp1 cig1* strains at half dose (2.5 × 10^7^ cells/mouse) and challenged with H99 (**D**) and the fungal burden of remaining survival animals at the endpoint of the experiment (**E**). *P < 0.05, ** *P* <0.005, ***P < 0.0005, and ****P < 0.0001 (log rank [Mantel-Cox] test). ns, no significance.

These findings demonstrate that Cig1-specific T cells are primed after challenge with *fbp1*∆, and this response is specific since immunization with *cig1*∆ failed to activate any Cig1-specific T cells. We then progressed to test the contribution of Cig1 in HK-*fbp1* vaccine protection against H99 wild-type challenge. Following our standard two-dose vaccination approach, we vaccinated mouse groups with either HK-*fbp1*, HK-*fbp1 cig1*, and a no-vaccination control (Fig. 7A). At a full dose (5 × 10^7^ cells/mouse) intranasal vaccination, we found there was no difference in survival following H99 challenge (Fig. 7B). The examination of fungal burden in the survival mouse lungs and brains at the endpoint showed that *HK-fbp1 cig1* vaccinated mice had modestly higher fungal CFU, but the difference was not statistically significant. There was no fungal CFU in the brains of both groups of mice (Fig. 7C). As the HK-*fbp1* vaccination is dose-dependent, as described in previous studies (9), we compared the two vaccinations again at half dose (2.5 × 10^7^ cells/mouse) to determine whether the difference in protection could be distinguished. Interestingly, at half-dose intranasal vaccination, we discerned that HK-*fbp1 cig1* had lower protection against H99 challenge compared to HK-*fbp1 vaccination* (Fig. 7D). Additionally, the fungal burdens in the survival mouse lungs and brains at the endpoint were determined. No difference was observed in lung fungal burden or dissemination to the brain in remaining survivors (Fig. 7E).

In aggregate, the results from these studies provide evidence that Cig1 is a novel Fbp1 substrate that contributes to the immunogenicity and elicited protection of HK-*fbp1* vaccination against *C. neoformans* challenge.

## DISCUSSION

In this study, we identified for the first time a single antigen, Cig1, that alters the immunogenicity of *fbp1*Δ, which contributes to the protection conferred by HK-*fbp1* vaccination against *C. neoformans* challenge. Furthermore, studies in this paper show that the capsule is required but not sufficient for HK-*fbp1* vaccine protection. Identifying the importance of the capsule in HK-*fbp1* builds on a growing consensus that capsule requirement for protection in several whole-cell *C. neoformans*-based vaccine candidates including *sgl1*∆ and Znf2^OE^ (15, 43). While other studies have revealed loss of capsule results in diminished or complete loss of protection, this present study supports that loss of the capsule directly alters the protective function of HK-*fbp1-*primed T cells noted by reduced production of key inflammatory cytokine IFN-γ. These findings emphasize that immunogenic antigens present on the cell surface or embedded in the capsule itself are critical for HK-*fbp1* protection as unmasking of several antigens present in the cell wall (i.e., β-glucans and chitin/chitosan) did not enhance protection. Immunofluorescent experiments with HK-*fbp1* vaccinated sera revealing antibodies binding to distinct inner regions of the *fbp1*Δ capsule supported this hypothesis.

The potential for mannoproteins as immunogens to be utilized in vaccination approaches has been described comprehensively in previous studies (36). Extensive mannosylation of mannoproteins is considered a primary contributor to enhanced immunogenicity and key to identification by phagocytes and stimulation of CD4^+^ T cells (23, 24). The role of chitin deacetylases (Cda1, Cda2, and Cda3) in maintaining cell wall integrity by modulating chitin to chitosan is a primary example of mannoprotein importance in fungal vaccine development (14, 31, 34, 44, 45). Although, in this Cda-deficient vaccination model, the enhanced immunogenicity is due more so to the loss of chitin deacetylase activity disturbing cell wall integrity rather than the immunogenicity of the enzymes themselves (14, 44). In the current study, we have ascertained that Cig1 is an overexpressed immunogenic antigen in *fbp1*Δ that contributes to eliciting protective inflammatory cytokines from HK-*fbp1* primed CD4^+^ T cells. In the wild-type parental strain H99, Cig1 is lowly expressed. This may be due to tight regulation so fungal cells can evade the host immune response.

The identification of mannoproteins as a contributor to the immunogenicity of *fbp1*Δ was a surprising but exciting finding. Our previous studies had ruled out several established fungal factors in the immunogenicity of *fbp1*Δ, including differences in mannoprotein levels, as measured by ConA staining (17). However, upon modification of the ConA staining protocol to reduce excessive background fluorescence, we saw clear differences in the ConA signal between H99 and the *fbp1*Δ mutant. We repeated this experiment for a total of six individual experiments and pooled all data in a cumulative analysis to confirm our initial findings. Additionally, this finding builds on a growing consensus that mannoproteins, including Cig1, contribute to the immunogenicity of now multiple fungal vaccine candidates (40). Nevertheless, this re-evaluation led to the current discovery of the Cig1 mannoprotein and its involvement in the Fbp1 E3 ligase pathway.

In this study, we identified that cryptococcal antigen Cig1 interacts with Fbp1. While we determined there is a direct protein-protein interaction between Cig1 and Fbp1, we unexpectedly identified that loss of Fbp1 also alters the transcriptional level of Cig1, as observed in qRT-PCR data. Fbp1 is part of the SCF E3 ligase complex that transiently interacts with substrate proteins set for degradation. We recently identified CDK-related kinase (Crk1) as a downstream substrate regulated by Fbp1 via its PEST domain sequence required for ubiquitin proteolysis (18). In the absence of Fbp1, Crk1 is accumulated and promotes the phosphorylation of Gpa1 G protein to contribute to “titan cell” formation. Furthermore, Crk1 stabilization by deletion of its PEST domain resulted in a similar phenotype to that of *fbp1*D, including virulence attenuation in murine survival and production of IFN-γ and IL-17 by CD4^+^ T cells (18). In our studies, we found that Cig1 is also upregulated in Crk1 overexpression strains (Crk1^OE^ and Crk1^ΔPEST^ strains). Previous studies have shown that the cyclic-AMP/protein kinase A (PKA) pathway can alter the cryptococcal capsule through regulation of pH-responsive transcription factor Rim101 (46, 47). Meanwhile, other studies have shown evidence to support the PKA pathway can alter the Ubiquitin Proteasome System (UPS) and function of the endoplasmic reticulum (48, 49) where mannoproteins are synthesized and later shuttled to the Golgi apparatus via the secretory system (49–51). From our results, we hypothesize a working model where regulation of Cig1 by Fbp1 occurs potentially through Crk1, and Rim101 may be a novel regulatory pathway at the transcriptional level, in addition to its Fbp1-mediated post-translational regulation. Future studies focused on confirming this potential pathway would be critical to understanding how loss of Fbp1 in the UPS directly impacts the expression of Cig1.

While these findings may reveal a novel regulatory pathway between Fbp1, Cig1, Crk1, and Rim101, the impact of *CIG1* deletion in the context of HK-*fbp1* vaccination is modest. The modest difference in vaccine protection between HK-*fbp1* and HK-*fbp1 cig1* vaccinated animals indicates that while Cig1 is a highly expressed immunogen in the *fbp1*Δ mutant, Cig1 alone is clearly not sufficient for HK-*fbp1* vaccine-induced protection. Considering the complexity of fungal immunogens, it is likely that *fbp1*∆-mediated immunogenicity involves multiple antigens in this mutant. Therefore, we acknowledge that Fbp1 likely regulates multiple different immunogenic factors, and Cig1 is one of them, which warrants further investigation.

In aggregate, our studies provide evidence that mannoprotein Cig1 and its presence embedded within the cryptococcal capsule plays an important role in shaping the immunogenicity of HK-*fbp1* vaccination-induced protection against *C. neoformans* challenge. These findings mark the first identification of an immunogenic antigen that directly contributes to protection in the HK-*fbp1* vaccination model. The recent report on mRNA-based vaccine using Cig1 as an antigen further confirms the immunogenicity of Cig1 as an important immunogen in *C. neoformans* (40). Furthermore, we have identified Fbp1 as a potential novel regulator of Cig1 through modulation of downstream substrates but remain to be further characterized. In conclusion, our data build on the growing body of knowledge of the relevance of mannoproteins as key targets for fungal vaccine development that may also play a role in other current fungal vaccine candidates.

## MATERIALS AND METHODS

### Animals and fungal cultures

Female mice with an average weight of 19 to 23 g were used throughout these studies. BALB/c and C57BL/6 genetic background of mice were purchased from the Jackson Laboratories. Animal studies were performed at the Public Health Research Institute Animal facility. All studies were conducted by following biosafety level 2 (BSL-2) protocols and procedures approved by the Institutional Animal Care and Use Committee (IACUC) and Institutional Biosafety Committee of Rutgers University. *Cryptococcus neoformans* clinical strain H99 and its mutants were cultured on the yeast extract-peptone-dextrose (YPD) medium.

### qRT-PCR

*Cryptococcus* cells from overnight culture were collected and washed two times with ddH_2_O, frozen down to −80°C for 6–8 hours, and lyophilized overnight to form powder. Total RNA extraction and first-strand cDNA synthesis were performed as described previously. Expressions of *CIG1, CMP1, CFO1, MP88, MP84, MP98, MP115,* and *GAPDH* were analyzed using SYBRGREEN advantage QPCR premix reagents (Takara). Gene expression levels were normalized using the endogenous control gene *GAPDH*, and the relative levels were determined using the comparative threshold cycle (*CT*) method. Quantitative real-time PCRs (qRT-PCRs) were performed using Aria MX Real Time PCR System (Agilent Technologies).

### Antibody binding assay

This protocol was conducted as previously described (15). Briefly, *Cryptococcus* cells were washed twice with sterile ddH_2_O and suspended in 4% formaldehyde and fixed for 5 min 2 × 10^7^ cells/mL at room temperature. Fixed cells were resuspended and blocked in 1% bovine serum albumin in 1X PBS at 4°C overnight. Cells were then washed twice with 1X PBS and resuspended in a 1:10 dilution of HK-fbp1 mouse serum in 1X PBS incubated at 4°C overnight. Cells were then washed the next day and resuspended in a 1:200 dilution of goat IgG IgM (H + L) anti-mouse secondary antibody conjugated to Alexa 488 (Invitrogen) at 4°C overnight. Following incubation, cells were washed twice with 1X PBS and co-stained with Calcofluor White and Indian Ink to then be imaged.

### Macrophage phagocytosis assays

Phagocytosis assays were performed in 96-well plates, using J774 macrophages at a concentration of 2.5 × 10^4^ cells/well that were allowed to double overnight at 37°C in activating medium (DME medium, 50 U/mL of IFN- γ, 1 g/mL of lipopolysaccharide [LPS]). Phosphate-buffered saline pH 7.4 (PBS)-washed fungal cells were opsonized with 20% mouse complement (Pel-Freez, AK) and added to the macrophages at an effector-to-target ratio of 1:2. Phagocytosis was allowed to occur for 2 h at 37°C in 10% CO_2_. The cells were then washed three times with 1X PBS and fixed with methanol at 4°C for 30 min. Giemsa stain was added to the wells at a dilution of 1:20, and the plates were incubated at room temperature for 30 min. The wells were washed once with 1X PBS and analyzed using an inverted microscope. For each well, three different fields were counted, for a total of at least 100 macrophages. The percent phagocytosis was determined by dividing the number of macrophages that contained *C. neoformans* by the total number of macrophages counted.

### Generation of tagged protein strains

The *CIG1* full-length gDNA was amplified with primers CX2373 and CX2375. The *CIG1* fragment was cloned into the BamHI/SpeI sites of a vector pCXU254 via the In-Fusion HD cloning kit (Takara). This cloning produced plasmid pCXU484 contains a *CIG1*:mCherry fusion that is under the actin promoter. The above plasmids were biolistically transformed into the H99 or *fbp1*∆ strain to generate strains CUX1454 and CUX1533, respectively.

### *In vivo* ubiquitination assay

CUX1454 (*P_ACT1_-CIG1:mCherry*) and CUX1533 (*fbp1*∆ *P_ACT_-CIG1:mCherry*) were cultured in YPD at a set OD_600_ 1.0 overnight. Cells were collected, washed, and cultured in YPD in the presence or absence of 20  µM MG-132 for 6  h. Total proteins were extracted in lysis buffer (50  mM Tris-HCl, 150  mM NaCl, 1% Triton X-100, 1  mM EDTA, 10  µM PMSF and 1 × EDTA free protease inhibitor). The Cig1 protein from the Cig1:mCherry total protein lysate was pulled down by Protein G Dynabeads (Invitrogen) bound to the rat anti-mCherry monoclonal antibody (Invitrogen) and eluted with 0.1 M glycine pH 2.0 and neutralized with 1.0M Tris HCL (pH 8.0). Accumulation of Cig1 polyubiquitination (Cig1-(Ub)n) was detected using anti-mCherry antibody (Invitrogen, M11217, 1:2000).

### Co-immunoprecipitation assay

Total proteins were purified and analyzed by immunoblotting with rabbit anti-FLAG (GeneScript) and rat anti-mCherry (Invitrogen) monoclonal antibodies. Proteins were pulled down by using anti-FLAG affinity gel (Sigma, A2220) incubated overnight and run on 12% SDS-PAGE gel. Samples were analyzed by immunoblotting with anti-FLAG antibody to detect Fbp1:FLAG or with anti-mCherry antibody to detect Cig1:mCherry.

### Vaccination strategy

*C. neoformans* mutant strains were heat-killed by following a previously described procedure (9). Briefly, fungal cells from YPD overnight cultures were spun-down and washed twice with sterile 1X PBS and resuspended and heated on a hot plate at 75°C for 105 min and then vortexed every 15 minutes to ensure proper killing of all cells. To ensure the killing of cells via heat treatment, the heat-killed cell suspension was plated on YPD agar plates and incubated at 30°C for 2 days. Mice were vaccinated intranasally in a 50 mL volume of either 5 × 10^7^ or 2.5 × 10^7^ heat-killed fungal cells after being anesthetized with a mix of ketamine (12.5 mg/mL) and xylazine (1 mg/mL) at day −42. Each group of 8 to 10 mice was boosted again with the same dose of heat-killed fungal strains at day −12. A group of unvaccinated mice was utilized as a control. Vaccinated and unvaccinated cohorts were challenged with 1 × 10^4^ live H99 cells via intranasal inoculation or other fungal species as specified below. Infected animals were weighed and monitored daily for disease progression, and mice displaying severe disease symptoms were euthanized. All survivors were euthanized on days 65–100 after challenge with live H99 cells, unless otherwise specified. Survival data from the murine experiments were statistically analyzed between paired groups by using the log rank (Mantel-Cox) test with PRISM version 10.0 (GraphPad Software, San Diego, CA) (*P* < 0.05 was considered statistically significant). The resulting data were plotted against time.

### Lung processing

Single-cell suspensions of pulmonary cells were prepared for flow cytometric analysis according to previously described methods (17). Briefly, the lung tissue was minced in 5 mL of 1X PBS containing 3 mg/mL collagenase type IV (Worthington). Samples were incubated at 37°C for 45 min and washed with 1X PBS three times. After digestion, residual RBCs were removed using RBC lysis buffer (155 mM NH_4_Cl and 10 mM NaHCO_3_, pH 7.2). Total numbers of lung cells collected for each sample were determined by counting numbers of cells on a hemocytometer. Lung single-cell suspensions were stained for CD4^+^ T cells with CD45 (30-F11 BUV395) and CD4^+^ (CD4 RM4-5, BV421) antibodies. CD8^+^ T cells were stained with CD8a 53-6.7 BV711 antibody. All antibodies used for lung staining were from BD Biosciences. All samples were analyzed using a BD LSR Fortessa flow cytometer and FlowJo software.

### Intracellular cytokine staining of T cells harvested in BALF and flow cytometry

For analyzing host immune responses, BALF samples were harvested at the endpoint after inoculation and prepared for flow cytometry analysis, as previously described (17). Briefly, BALF was collected in 5 mL of 1x PBS buffer using a catheter inserted into the trachea of animal post-euthanasia. Reb blood cells (RBCs) were removed using RBC lysis buffer. BALF cells were then plated in a 96-well round-bottom plate and restimulated using BD-leukocyte activation cocktail containing BD GolgiPlug (BD Biosciences) according to the manufacturer’s instructions. Six hours after activation, BALF cells were surface-stained with fluorescently labeled antibodies against Thy1.2, CD4, and CD8. Samples were fixed in 1% paraformaldehyde overnight. Prior to intracellular staining, the samples were permeabilized with 1X BD Perm/Wash buffer according to the manufacturer’s instructions. Intracellular cytokine staining (ICCS) was done using fluorescently labeled antibodies against IFN-γ, IL-17A, TNF-a, and IL-13 diluted in 1X BD Perm/Wash for 30 min on ice. Cells were cell surface-stained for T cells with Thy1.2 (53-2.1 PE-Cy7), CD4 (RM4-5 BV421), CD8 (53-6.7 BV711), and ICCS for IFN-g (XMG1.2 PE), IL-17A (eBio17B7 APC), TNF-a (MP6-XT22 Alexa Flour 700), and IL-13 (eBio13A FITC).

### Lung lymphocyte *ex vivo* restimulation

This protocol was used and modified based on the previously established protocol by Weisner and colleagues (52). Lung samples were minced in 5 mL of 1X PBS containing 3 mg/mL collagenase type IV (Worthington) and incubated at 37°C for 45 min. Samples were then washed with 1X PBS and then resuspended in 40% Percoll (Sigma) layered on top of 66% Percoll and centrifuged at 2,000 rpm for 20 min to purify lung immune cells. Single-cell lung suspension samples of single dose vaccinated mice were pooled together (*n* = 10) incubated on a 48 well plate with CD28 1:100 dilution (Biolegend clone 37.51) and antigen for 2 hours incubated at 37°C with 5% CO_2_. After restimulation with the antigen, samples were incubated with 1X Brefeldin A (Invitrogen) for 4 hours at 37°C with 5% CO_2_. Lung lymphocytes were then stained and fixed for overnight intracellular cytokine staining with the Foxp3 fix/permeabilization kit (eBioscience) and later analyzed via flow cytometry. Activated CD4^+^ T cells' production of cytokines was gated as near-IR LIVE/DEAD (Invitrogen)^−^, CD90^+^ (SE10 BV785), NK1.1^−^ (PK136 AF700), Ly6C^−^ (AL-21 FITC), Ly6G^−^ (IA8 IL-17A), SiglecF^−^ (E50-2440 BV421), CD11c^−^ (N418 AF700), TCRb^+^ (H57-597 PE/CYNANINE7), TCRgd^−^ (GL3 PE/CYNANINE5.5), CD8^−^ (S3-6.7 BV605), CD4^+^ (GK1.5 BUV395), and CD44^+^ (NIMR8 FITC).

### CD4^+^ T cell isolation and CD4^+^ T cell recall response

Lung-draining mediastinal lymph nodes (mLNs) were collected and placed in 10 mL of 1X PBS. Total lymphocyte cell suspensions were prepared by gently releasing the cells into the 1X PBS by grating the lymph nodes with the frosted ends of two glass slides. Individual samples from each group were pooled (5 to 6 mice). CD4^+^ T cells were purified using a negative-sorting CD4^+^ isolation kit (Miltenyi Biotec, Inc., Auburn, CA). CD4^+^ T cell isolation was done by following the manufacturer’s instructions and was consistently found to be 90% pure, as assessed by flow cytometry. Purified CD4^+^ T cells (2 × 10^5^) were cultured with T cell-depleted antigen-presenting cells (3 × 10^5^) in RPMI containing 10% fetal calf serum (FCS) and penicillin-streptomycin (2,200 U/mL; Gibco). The cultures were plated in flat-bottom 96-well plates and incubated at 37°C with 5% CO_2_ for 72 h. Antigen-presenting cells were prepared from the spleen of syngeneic, uninfected donor mice. In brief, splenic cell suspensions were depleted of T cells by antibody complement-mediated lysis. Splenic cells were incubated with anti-Thy1.2 antibodies and rabbit complement (Low Tox; Cedarlane Labs, Hornby, ON, Canada) at 37°C for 60 min. To measure *Cryptococcus*-specific responses, CD4^+^ antigen-presenting cell cultures were incubated with sonicated H99 as a source of fungal antigens. The amount of antigen used was adjusted to a multiplicity of infection of 1:1.5 (antigen-presenting cell:yeast). The fungal growth inhibitor voriconazole was used at a final concentration of 0.5 mg/mL to prevent any fungal cell outgrowth during the culture period. After 72 h post culture, with initiation at 37°C with 5% CO_2_, supernatants were collected for cytokine analysis by enzyme-linked immunosorbent assay (IL-2, IL-5, IL-17, TNF-a, and IFN-γ, Invitrogen) by following the manufacturers’ instructions.

### Fungal burdens in infected organs

Infected animals were sacrificed at designated time points and the endpoint of the experiment according to the Rutgers Institutional Animal Care and Use Committee (IACUC)-approved animal protocol. Infected lungs, brains, and spleens were also isolated and homogenized in 3 mL of 1X PBS. Resuspensions were diluted, 200 uL of each dilution was spread on YPD medium with ampicillin and chloramphenicol, and numbers of colonies were determined after 3 days of incubation.

### Statistics

GraphPad Prism version 10.2.2 (GraphPad Software, La Jolla, CA) was used for statistical analyses and graph design. Survival data from the murine experiments were statistically analyzed between paired groups by using the log rank (Mantel-Cox) test where *P* values of < 0.05 were considered statistically significant. Statistical analysis of *in vivo* and *in vitro* experiments with three or more groups, Shapiro-Wilk test for normality, and Brown-Forsythe test for equal variance among groups were performed. For multiple-comparison analysis of three or more groups that met normality and equal variance conditions, a one-Way ANOVA with Dunnett’s multiple comparisons test or Kruskal-Wallis test with Dunn’s multiple comparison test was used.

## SUPPLEMENTAL MATERIAL

Supplemental material iai.00355-25-s0001.docx Fig. S1 to S4; Tables S1 and S2.

ASM does not own the copyrights to Supplemental Material that may be linked to, or accessed through, an article. The authors have granted ASM a non-exclusive, world-wide license to publish the Supplemental Material files. Please contact the corresponding author directly for reuse.
