# Supplementary material for: Mannoprotein Cig1 contributes to the immunogenicity of a heat-killed F-box protein Fbp1 Cryptococcus neoformans vaccine model
Source: Infect Immun. 2025 Nov 28;94(1):e00355-25. doi: 10.1128/iai.00355-25 (PMC12797982; doi:10.1128/iai.00355-25)
Supplement: Supplemental material — Fig. S1 to S4; Tables S1 and S2. [file iai.00355-25-s0001.docx]

**Supplementary materials:**

**Mannoprotein Cig1 Contributes to the Immunogenicity of a Heat-Killed F-box Protein Fbp1 *Cryptococcus neoformans* Vaccine Model**

Samantha Avina^1,2^, Siddhi Pawar^1,2^, Roshni N. Kadam^1,2^, Amariliz Rivera^3^, Chaoyang Xue^2^

^1^School of Graduate Studies at the Health Science Campus, New Jersey Medical School, Rutgers University Newark, New Jersey, USA

^2^Public Health Research Institute, and Department of Microbiology, Biochemistry, and Molecular Genetics, New Jersey Medical School, Rutgers University, Newark, New Jersey, USA

^3^Center for Immunity and Inflammation, and Department of Pediatrics New Jersey Medical School, Rutgers University Newark, New Jersey, USA

**Supplemental figures**

**
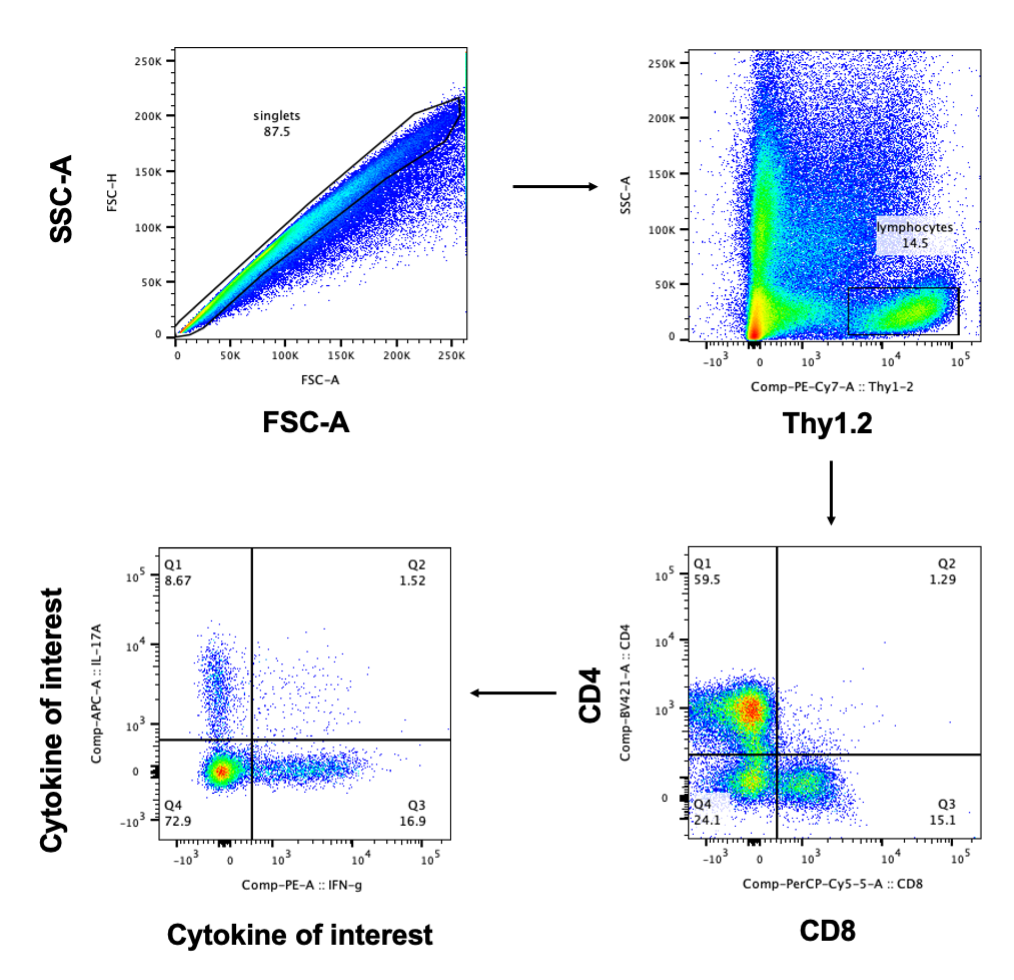
**

**Figure S1. Bronchoalveolar lavage fluid (BALF) CD4^+^ T cell isolation gating strategy.**

**
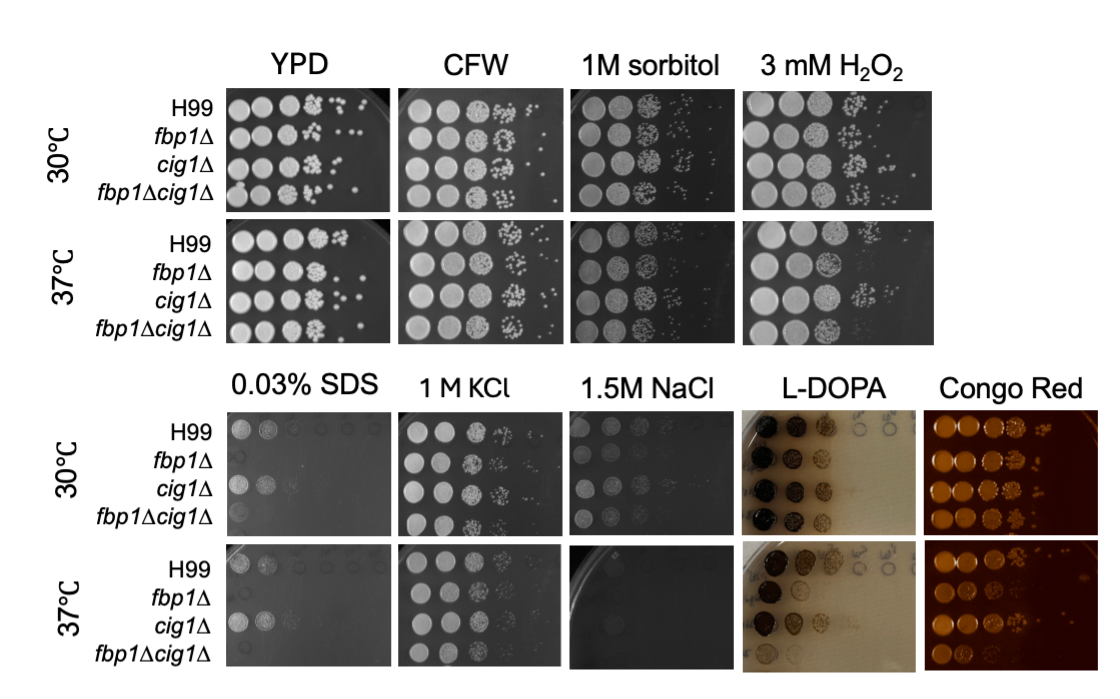
**

**Figure S2. Cig1 mannoprotein is upregulated in *fbp1∆* and alters *Cn* cell wall integrity.** Comparison of parental H99, *fbp1***∆*,*** *cig1***∆*,*** and *fbp1*∆ *cig1*∆ mutant strains on different growth conditions at 30°C and 37°C.


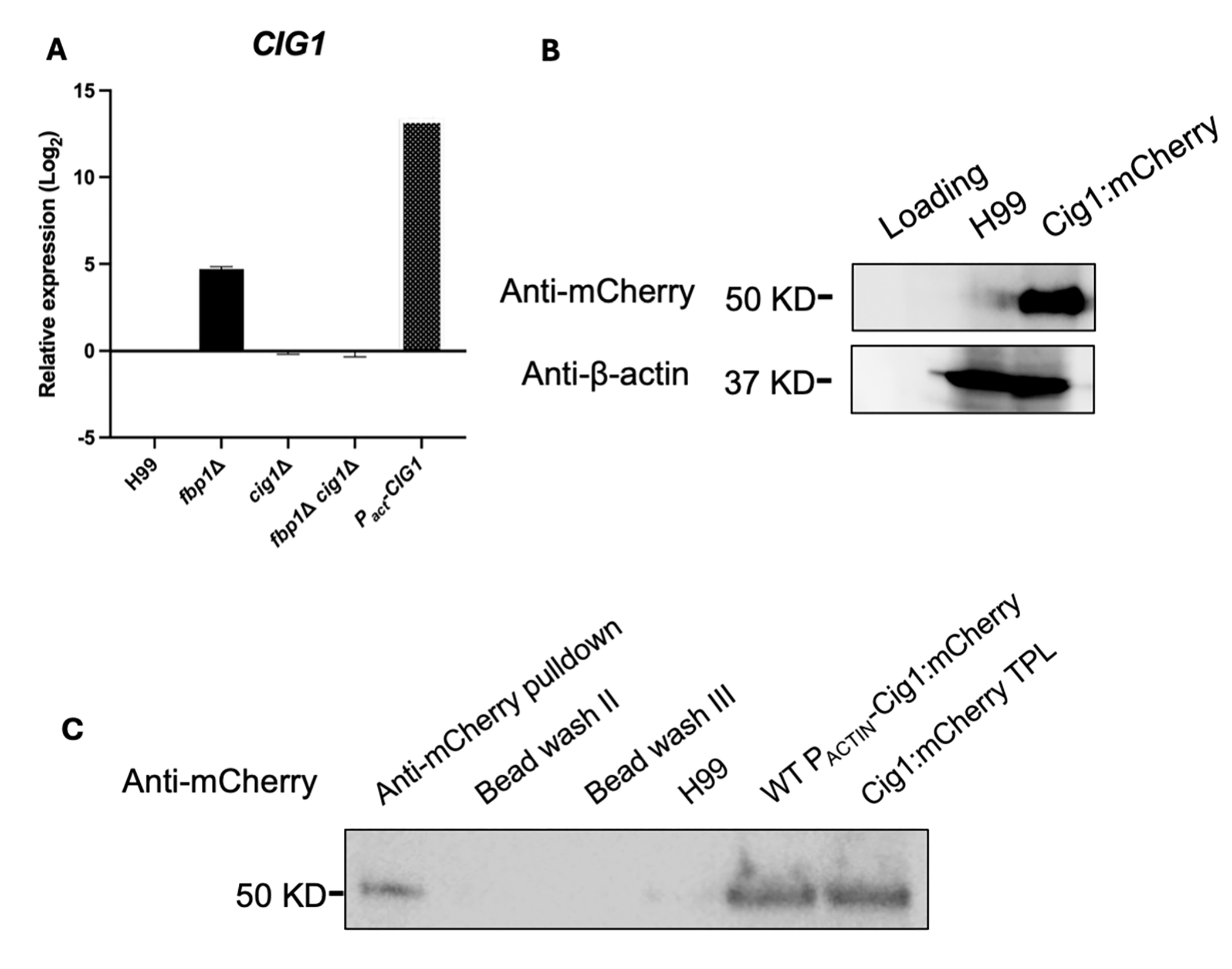


**Figure S3. Construction and validation of *P_ACTIN_-CIG1:*mCherry strain for Cig1 protein enrichment. A**. qRT-PCR of Cig1 expression in mutant strains. **B.** Western blot analysis of Cig1-mCherry tagged protein with β-actin as a control. **C.** Protein enrichment of Cig1 from *P_ACTIN_-CIG1:*mCherry expressing *C. neoformans* total protein lysate via co-IP with rat anti-mCherry antibody bound to protein G Dynabeads (Invitrogen).

**
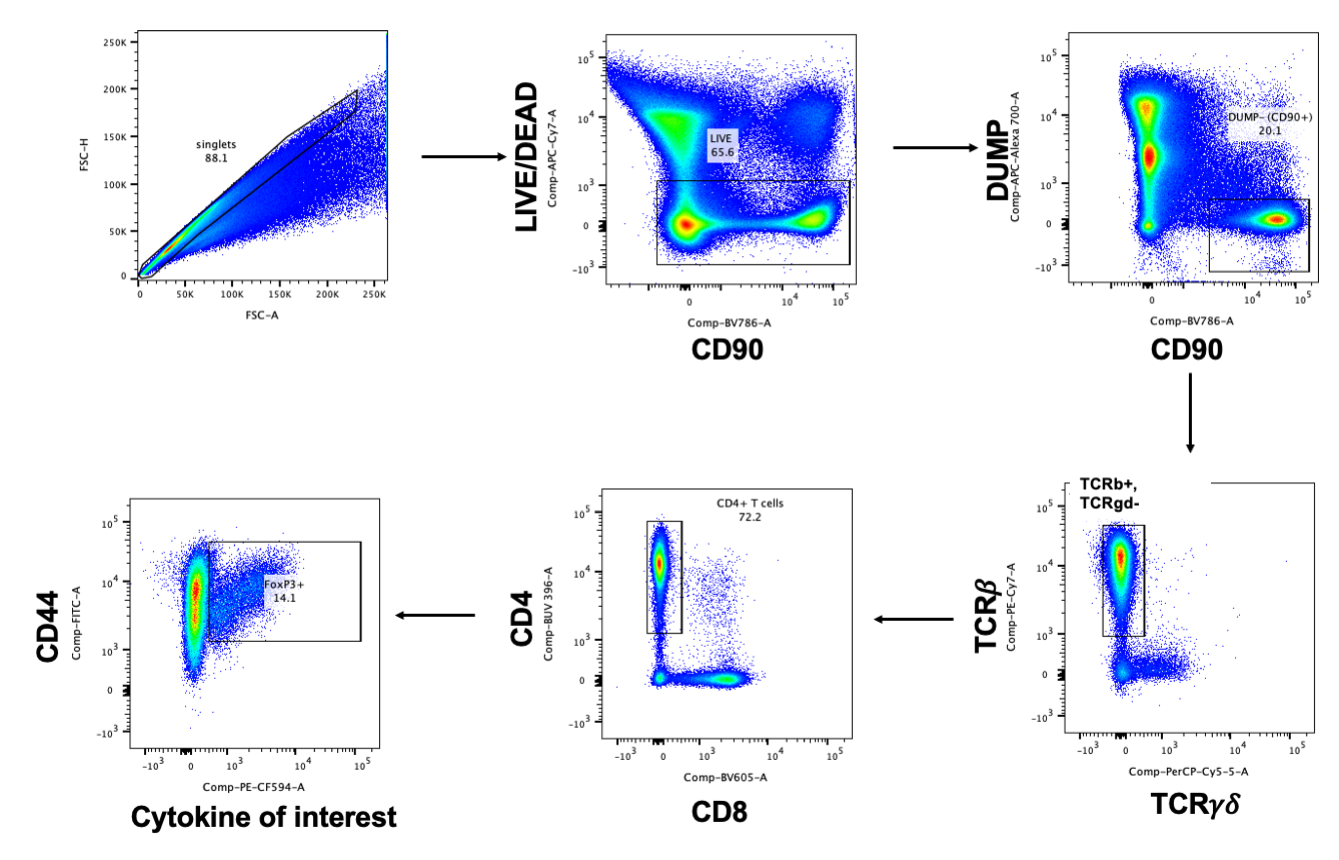
**

**Figure S4. CD4^+^ T cell purification gating strategy in ex vivo antigen restimulation experiment.**

**Supplementary table 1: List of strains and plasmids used in this study.**

| **strains** | **genotype** | **Source/reference** |
| --- | --- | --- |
| H99 | MAT**α** | (1) |
| KN99a | MAT**a** | (2) |
| CUX2 | MAT**α** *fbp1*∆::*NEO* | (3) |
| CUX1446 | MAT**α** *cig1*∆*::NAT* | Kronstad, J. (4) |
| CUX1454 | MAT**α** *P_ACT1_-CIG1:mCherry-NAT* | This study |
| CUX1455 | MAT**α** *fbp1*∆::*NEO cig1*∆::*NAT* | This study |
| CUX1459 | MAT⍺ *fbp1∆*::*NEO* *ura5 P_ACT1_-FBP1:FLAG-URA5 P_ACT1_-CIG1:mCherry-NAT* | This study |
| CUX1533 | MAT**α** *fbp1*∆::*NEO P_ACT1_-CIG1:mCherry-NAT* | This study |
| CUX1291 | MAT**α** *P_ACT1_-CRK1:mCherry-NAT* | (3) |
| CUX1293 | MAT**α** *P_ACT1_-CRK1^ΔPEST^:mCherry-NAT* | (3) |
| **Plasmids** | **Description** | **Source/reference** |
| pCXU254 | P_ACT1_-*CHO1*:mCherry*-NAT* | (5) |
| pCXU484 | *P_ACT1_-CIG1:mCherry-NAT* | This study |

**References**

1. Perfect JR, Schell WA, Rinaldi MG. 1993. Uncommon invasive fungal pathogens in the acquired immunodeficiency syndrome. Journal of Medical Veterinary Mycology 31:175-9.

2. Nielsen K, Cox GM, Wang P, Toffaletti DL, Perfect JR, Heitman J. 2003. Sexual cycle of Cryptococcus neoformans var. grubii and virulence of congenic a and alpha isolates. Infection and Immunity 71:4831-41.

3. Cao C, Wang K, Wang Y, Liu T-B, Rivera A, Xue C. 2022. Ubiquitin proteolysis of a CDK-related kinase regulates titan cell formation and virulence in the fungal pathogen *Cryptococcus neoformans*. Nature Communications 13:6397.

4. Cadieux B, Lian T, Hu G, Wang J, Biondo C, Teti G, Liu V, Murphy MEP, Creagh AL, Kronstad JW. 2013. The mannoprotein Cig1 supports iron acquisition from heme and virulence in the pathogenic fungus *Cryptococcus neoformans*. The Journal of Infectious Diseases 207:1339-1347.

5. Konarzewska P, Wang Y, Han GS, Goh KJ, Gao YG, Carman GM, Xue C. 2019. Phosphatidylserine synthesis is essential for viability of the human fungal pathogen Cryptococcus neoformans. Journal Biological Chemistry 294:2329-2339.

**Supplementary table 2: List of primers used in this study.**

| Primers | Sequences (5’-3’) | References/Note |
| --- | --- | --- |
| CX49 | TGAGAAGGACCCTGCCAACA | GAPDH F qPCR |
| CX50 | ACTCCGGCTTGTAGGCATCAA | GAPDH R qPCR |
| CX18993 | GACGACATCTATAGCAGATC | MAT alpha F |
| CX18994 | CCAAAAGCTGATGCTGTGGA | MAT alpha R |
| CX18995 | TCCACTGGCAACCCTGCGAG | MAT **a F** |
| CX18996 | ATCAGAGACAGAGGAGCAAGAC | MAT **a R** |
| CX284 | GGAATCAACGAAGATCTGAAGG | FBP1 F positive |
| CX1104 | TGTGGATGCTGGCGGAGGATA | FBP1 R positive |
| CX282 | GACCGTCAAGAAGCAACTCG | FBP1 F negative |
| CX283 | CAGATTGATAGCTTGCAGCTTC | FBP1 R negative |
| CX2259 | CCTCGTGGTTTCTCAGCTTC | CIG1 F qPCR |
| CX2260 | CCACCGCTCATAGGGTAAGA | CIG1 R qPCR |
| CX2261 | ACATGACCGCCCTCAGTAAC | MP98 F qPCR |
| CX2262 | CCTCGGCAATCATACGAACT | MP98 R qPCR |
| CX2263 | TTCGAGGCTGGAAAGTCCTA | CFO1 F qPCR |
| CX2264 | ACTTCGACACCGTCCATTTC | CFO1 R qPCR |
| CX2265 | ACCTACCATGCTTCCCCTCT | CMP1 F qPCR |
| CX2266 | GTCGGAGGACTGGTTGACAT | CMP1 R qPCR |
| CX2267 | GAATGCCAGCACACTCTTGA | MP88 F qPCR |
| CX2268 | GCTGCTGGAAGGTAGAGGTG | MP88 R qPCR |
| CX2269 | GCGTTGAGGCTTACTTCGAC | MP84 F qPCR |
| CX2270 | GTTAAAAGCATCGGCCACAT | MP84 R qPCR |
| CX2271 | CGCCTTGAGTGCAGACAATA | MP115 F qPCR |
| CX2272 | TGTTGCGGGAAAGGATAGTC | MP115 R qPCR |
| CX2373 | CAACATGTCTGGATCCATGATTTTTAATCGTTTCACATTCA | CIG1 F infusion pCXU254/BamHI |
| CX2375 | CCATTCTAGAACTAGTGAGACGCTCCTTGGTGGGT | CIG1 R infusion pCXU254/SpeI |
